# Task-dependent effects of nicotine treatment on auditory performance in young-adult and elderly human nonsmokers

**DOI:** 10.1038/s41598-021-92588-z

**Published:** 2021-06-23

**Authors:** Shuping Sun, Michelle R. Kapolowicz, Matthew Richardson, Raju Metherate, Fan-Gang Zeng

**Affiliations:** 1 Department of Otolaryngology – Head and Neck Surgery, The First Affiliated Hospital, Zhengzhou University, Zhengzhou, 450052 Henan China; 2 Center for Hearing Research, University of California Irvine, Irvine, CA USA

**Keywords:** Neuroscience, Physiology, Psychology, Medical research

## Abstract

Electrophysiological studies show that nicotine enhances neural responses to characteristic frequency stimuli. Previous behavioral studies partially corroborate these findings in young adults, showing that nicotine selectively enhances auditory processing in difficult listening conditions. The present work extended previous work to include both young and older adults and assessed the nicotine effect on sound frequency and intensity discrimination. Hypotheses were that nicotine improves auditory performance and that the degree of improvement is inversely proportional to baseline performance. Young (19–23 years old) normal-hearing nonsmokers and elderly (61–80) nonsmokers with normal hearing between 500 and 2000 Hz received nicotine gum (6 mg) or placebo gum in a single-blind, randomized crossover design. Participants performed three experiments (frequency discrimination, frequency modulation identification, and intensity discrimination) before and after treatment. The perceptual differences were analyzed between pre- and post-treatment, as well as between post-treatment nicotine and placebo conditions as a function of pre-treatment baseline performance. Compared to pre-treatment performance, nicotine significantly improved frequency discrimination. Compared to placebo, nicotine significantly improved performance for intensity discrimination, and the improvement was more pronounced in the elderly with lower baseline performance. Nicotine had no effect on frequency modulation identification. Nicotine effects are task-dependent, reflecting possible interplays of subjects, tasks and neural mechanisms.

## Introduction

Nicotine, an exogenous agonist for nicotinic acetylcholine receptors, has been shown to improve cognitive functions such as attention, learning, and memory for healthy young adults^1,2^, healthy older adults^3^, and those with dementia^4^. In the mouse auditory cortex, systemic nicotine sharpens receptive fields at characteristic frequencies while suppressing spectrally distant, non-characteristic frequency responses^5–7^. Conversely, mice lacking β2-containing nicotinic acetylcholine receptors, the predominant receptor subtype in forebrain, exhibit impaired auditory discrimination^8^. These results suggest that nicotine aids with auditory gating in order to facilitate processing of relevant sounds while filtering out irrelevant sounds^9^. Human electrophysiological studies did not find consistent evidence for the stimulus-filter model in auditory processing^10,11^. Human behavioral studies in younger adults found that nicotinic effects depend upon task difficulty, with a greater benefit in more difficult conditions, such as tone-in-noise detection and auditory selective attention tasks^11,12^.

It is possible that older individuals may derive a greater benefit from nicotine treatment compared to their younger counterparts because older adults often have difficulty with speech understanding even with little to no hearing loss^13,14^. This difficulty, which cannot be overcome by traditional hearing aids or cochlear implants, is related to suprathreshold impairments in both peripheral and central processing^15–18^. To the extent that these behavioral impairments reflect the age-related changes in nicotinic acetylcholine receptor signaling^19–21^, nicotine treatment may improve auditory performance in the elderly.

The present study selected two frequency tasks that rely on information processing over a relatively broad frequency region^22^ and one intensity task that relies on information processing at the characteristic frequency^23^. Both young and elderly participants, all healthy nonsmokers, were evaluated for these three tasks before and after treatment with either nicotine or placebo. The primary hypothesis was that, relative to placebo, nicotine treatment would improve auditory performance. The secondary hypothesis was that individuals with lower pre-treatment baseline performance would obtain a greater benefit from nicotine treatment^24–26^.

## Materials and methods

### Subjects

Twenty individuals participated in the study: 10 young adults (age range = 19–23, *M* ± *SD* = 21 ± 4 years, 5 females) and 10 elderly adults (age range = 61–80, *M* = 69 ± 6, 5 females). Participants gave written informed consent approved by the University of California Irvine’s Institutional Review Board and were monetarily compensated. The methods were in accordance with principles set forth in the Belmont Report and Declaration of Helsinki. Participants were screened prior to the start of the study to ensure no known severe hearing dysfunction, medical, or mental health illnesses. Participants were also given hearing tests to screen for hearing loss. Young adult participants had normal hearing with thresholds ≤ 20 dB HL (Hearing Level) at octave frequencies between 0.125 and 8 kHz. Except for one who had mild hearing of 30–35 dB HL at 125 and 250 Hz, all elderly participants had normal hearing (≤ 20 dB HL) at octave frequencies between 0.125 and 2 kHz. On average, the elderly participants had mild-to-moderate hearing loss at 4 kHz (33 ± 21 dB HL) and 8 kHz (45 ± 24 dB HL). To minimize the influence of hearing loss, all three tasks had stimulus frequencies below 2 kHz (see “Experimental protocol” below).

To ensure little to no nicotine dependence from use or exposure, participants were required to have a score of 0–2 out of 10 on the Fagerstrӧm index of smoking dependency^27,28^. Eighteen participants were non- or social-smokers, defined as smoking no more than 100 cigarettes in their lifetime and none in the past year^24^. Two subjects reported quitting smoking more than 20 years ago. To avoid chemical interactions, participants were instructed to abstain from alcohol consumption for 24 h and food consumption for ≥ 1 h prior to testing. To avoid caffeine withdrawal in regular caffeine consumers, ½ cup of a caffeine-containing beverage was permitted ≥ 1 h prior to testing^29^.

### Experimental protocol

Three tasks were selected for their lower performance in older than younger adults, including frequency and intensity discrimination^17^ and frequency modulation identification^18^. The previously described experimental protocols were closely followed in the present study. Briefly, the frequency discrimination experiment measured the just-noticeable-difference in frequency for a 400-ms, 500-Hz pure tone presented at 55 dB SPL. The intensity discrimination experiment measured the just-noticeable-difference in loudness for a 400-ms, 500-Hz pure tone presented at 55 dB SPL. The just-noticeable-difference was obtained by a three-interval, two-alternative, forced-choice adaptive procedure using a two-down, one-up rule to yield 71% correct performance^30^. The just-noticeable-difference in Hz was logarithmically transformed to conform to a normal distribution.

The frequency modulation identification experiment measured the signal-to-noise ratio required to identify an up-down or down-up frequency modulation “signal” in the presence of a ‘noise”^18^. The 400-ms signal consisted of harmonics with a fundamental frequency of 189 Hz and a single spectral peak or formant at 1000 Hz. The formant frequency was dynamically modulated by a 5-Hz triangular wave with a 55% modulation swing. The 800-ms noise also consisted of similarly-modulated harmonics but with a different fundamental frequency of 107 Hz. The signal was presented at the temporal center of the noise. The signal level was constant at 55 dB SPL, while the noise was adaptively varied. The participant had to report whether the modulation was up-down or down-up in a single-interval, two-alternative, forced-choice task. A three-down, one-up rule estimated the signal-to-noise ratio, at which the participant correctly identified 79% of the frequency modulated signal^31^.

All experiments took place in a double-walled, sound-attenuated booth. The 55-dB SPL was chosen because previous studies^17^ found that the performance difference between young and elderly listeners is greater at lower than higher levels. Given that the present subjects had a threshold of 10–15 dB HL (= 25–30 dB SPL) at 500 and 1000 Hz, 55 dB SPL was a reasonable choice, presenting the stimuli at 25–30 dB SL, which sounded relatively soft but clearly audible. Stimuli were presented binaurally via a sound card (Creative Labs E-MU 0404 USB digital audio system, Creative Technology Ltd., Singapore, 16-bit, 44.1 kHz), through circumaural headphones (Sennheiser HAD-200, Wedemark, Germany). The reported result for each participant was the arithmetic mean of the estimate obtained in three to five runs. In all three tasks, lower values reflected better performance.

### Study design

All procedures leading up to experiments, including method of drug delivery and time between sessions to allow for drug clearance followed those specified by Pham et al.^12^. Briefly, six mg of nicotine was delivered in the form of two pieces of mint-flavored polacrilex gum (4 mg and 2 mg; Nicorette, Johnson & Johnson, Inc). Two mint-flavored gum (Eclipse), resembling the nicotine gum in size, shape, color, and texture, served as the placebo. Furthermore, subjects wore a blindfold during both administrations to mask any potential visual differences between placebo and nicotine gums; a drop of Tabasco sauce was added to each gum piece to disguise taste bias^32^. Test occurred between 8:00 am and 6:00 pm and took place at a consistent time across sessions to avoid confounding arousal and attention effects. All three experiments were completed in two sessions, in which a treatment (nicotine or placebo) was given while pre- and post-treatment data were collected. Audiograms were measured prior to pre-treatment testing in the first session. Afterwards, participants received either nicotine or placebo gum in a randomized design. The protocol was repeated with the alternate treatment (nicotine or placebo) in the second session adhering to a single-blind intra-subject design. Treatment sessions were separated by ≥ 48 h to allow for treatment clearance. Timing was carefully controlled to assure that nicotine plasma concentration at this dosage reached and maintained peak levels for the duration of the three experiments^12,33^.

### Pulse oximetry, mood changes, and side effects

Neither nicotine nor placebo treatment significantly changed blood oxygen saturation in either group (mean ± SD: *young*, pre-nicotine 97.9 ± 0.3%; post-nicotine 98.2 ± 0.6; pre-placebo 97.9 ± 0.9; post-placebo 98.1 ± 1.1; *elderly*, pre-nicotine 96.0 ± 1.8; post-nicotine 96.8 ± 1.5; pre-placebo 97.0 ± 1.8; post-placebo 97.0 ± 1.0). Nicotine treatment did not significantly change pulse rate in either group (*young*, pre-nicotine 71.0 ± 4.5/min; post-nicotine 73.9 ± 8.1; *elderly*, pre-nicotine 71.0 ± 8.2; post-nicotine 70.6 ± 6.4). Placebo treatment did not significantly alter pulse rate for the young (pre-placebo 70.5 ± 8.2; post-placebo 68.8 ± 8.5) but significantly decreased pulse rate for the elderly (pre-placebo 72.2 ± 8.2; post-placebo 66.6 ± 8.0, p < 0.01). The reason for the decreased pulse rate was not clear.

Participants also provided subjective pre- and post-treatment ratings using a 9-category mood profile, where responses for each category were binary (e.g., tense/relaxed) and using a 5-point side effects scale, where 1 corresponded to no symptoms and 5 corresponded to severe symptoms such as jittery, headache, nausea, or vomiting^10^. Ratings were averaged across the three experiments. No significant pre- versus post-treatment change in mood was found for nicotine or placebo treatment in either group (both *p* > 0.05). No post-treatment difference between placebo and nicotine was observed in either group for ratings of side effects (both *p* > 0.05).

### Data analysis

Mixed 2 × 2 × 2 ANOVA was used to examine the effects of age (young vs. elderly), treatment (nicotine vs. placebo), and time (pre vs post), with age being a between-subjects factor, and treatment and time being within-subjects factors. For any significant factor or interactions, effect sizes were reported as eta squared or *η*^2^ (small effect: ~ 0.01, medium effect: ~ 0.06, large effect: ~ 0.14). To test the primary hypothesis that nicotine improves auditory performance, two-tailed, two-sample *t*-tests were used to test if combined (i.e., young and elderly) post-nicotine minus placebo scores were significantly different from zero, and effect sizes were determined with Cohen’s *d* (small effect: ~ 0.2, medium effect: ~ 0.5, large effect: ~ 0.8). To test the secondary hypothesis that participants with lower baseline performance would benefit more from nicotine treatment, a linear regression was conducted between the nicotine-placebo post-treatment difference and the baseline performance for each of the three auditory tasks. Baseline performance was the average of the two sets of pre-treatment data from nicotine and placebo conditions. Justification for combining pre-treatment data was performed via a Kolmogorov–Smirnov test, revealing that the combined data follow a normal distribution for each experiment (frequency discrimination, *p* = 0.34; frequency modulation identification, *p* = 0.83; intensity discrimination, *p* = 0.83).

## Results

### Task-dependent nicotine effects

**Figure 1:**
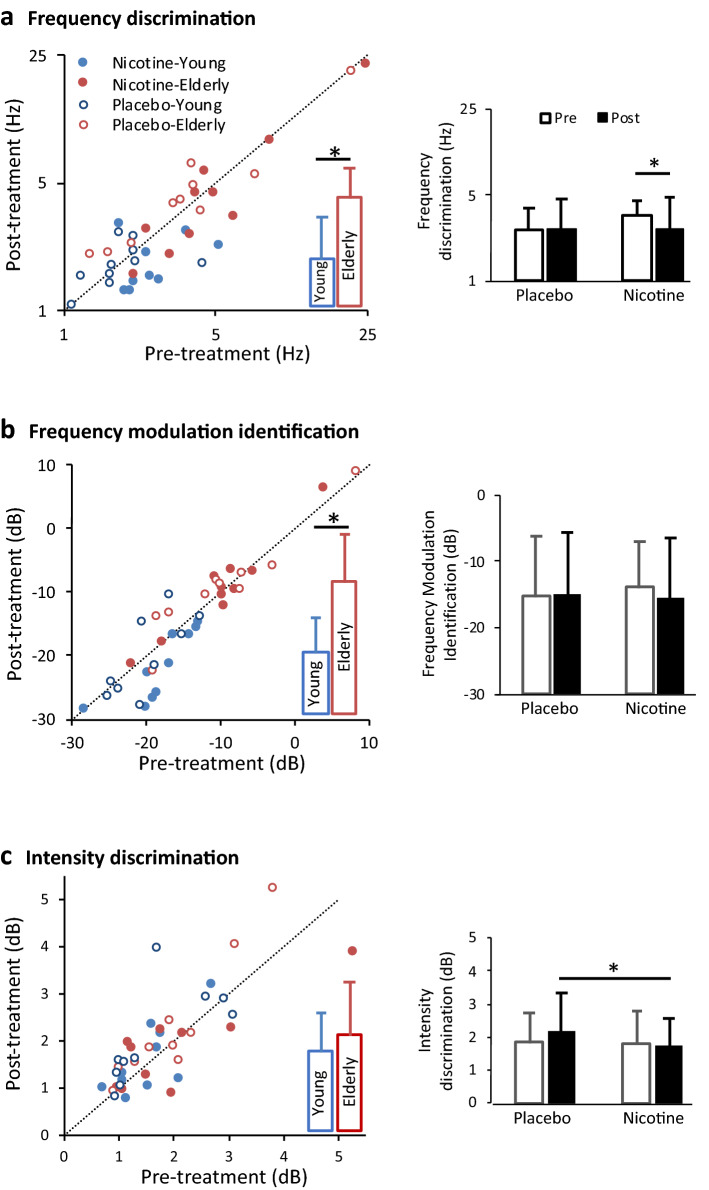
Post- versus pre-treatment performance for three auditory tasks. **(a)** Frequency discrimination thresholds at 500 Hz. The left panel shows individual results from both nicotine (filled circles) and placebo (open circles) conditions for young (blue) and elderly adults (red). Data points below the diagonal line indicate improved post-treatment performance, whereas points above denote worsened performance. The insert in the left panel shows between-subjects comparisons between young (blue bar) and elderly (red bar) adults. The right panel shows 2 × 2 within-subjects comparisons between the treatment (nicotine vs. placebo on the x-axis) and time (pre- vs. post-treatment = open vs. filled bars) factors. Error bars represent one standard deviation. The asterisk above the line indicates a significant difference between the two conditions. **(b)** Frequency modulation identification results are displayed following the same convention as (**a). (c)** Intensity discrimination results are displayed following the same convention as (**a).**

Figure 1 contrasts pre- versus post-treatment results from the three tasks. Figure 1a (left panel) shows frequency discrimination results from both nicotine (filled circles) and placebo (open circles) conditions for young (blue) and elderly adults (red). Data points below the diagonal line indicate improved post-treatment performance, whereas those above indicate worsened performance. Overall, frequency discrimination was significantly worse (i.e., greater) for the elderly (red bar) than younger (blue bar) individuals (see insert in the left panel: 4.2 ± 2.0 Hz vs. 1.9 ± 1.5 Hz; *F*(1,18) = 10.66, *p* = 0.004, *η*^2^ = 0.37). This age-related difference replicates the previous result^17^. Within-subjects comparisons (right panel) found a borderline significant effect for both treatment [*F*(1,18) = 4.12, *p* = 0.057] and time [*F*(1,18) = 4.18, *p* = 0.056], but a significant interaction between the two factors [*F*(1,18) = 13.22, *p* = 0.002, *η*^2^ = 0.78]. The significant interaction was that nicotine significantly improved frequency discrimination by 24% over the pre-treatment baseline (3.4 ± 2.0 Hz vs. 2.6 ± 2.1 Hz, open bar vs. filled bar above “Nicotine”). There was no significant three-way interaction.

Figure 1b uses the same format to display frequency modulation identification results. Similar to the previous result^18^, younger individuals outperformed elderly individuals by about 10 dB (− 19.5 ± 5.1 dB vs. − 9.7 ± 7.4 dB; *F*(1,18) = 15.77, *p* = 0.001, *η*^2^ = 0.96). Within-subjects comparisons found no significant effect for either treatment or time, nor was there any significant interaction between the two factors [F(1,18) < 3.29, *p* > 0.09]. The three-way interaction was not significant.

Figure 1c displays intensity discrimination results. Different from the previous significant result^17^, the present study found no difference between the elderly and younger individuals [F(1,18) = 0.71, *p* = 0.41]. Within-subjects comparisons found a significant treatment effect [*F*(1,18) = 5.37, *p* = 0.03, *η*^2^ = 0.23] but no time effect [*F*(1,18) = 1.69, *p* = 0.21] nor any significant interaction [*F*(1,18) = 2.60, *p* = 0.12]. Different from the frequency discrimination result, the significant nicotine effect here was due to the 29% worsened post-treatment performance in the placebo condition (2.2 ± 1.2 dB vs. 1.7 ± 0.8 dB, the two filled bars in the right panel). There was no significant three-way interaction.

### Post-treatment performance as a function of baseline

**Figure 2:**
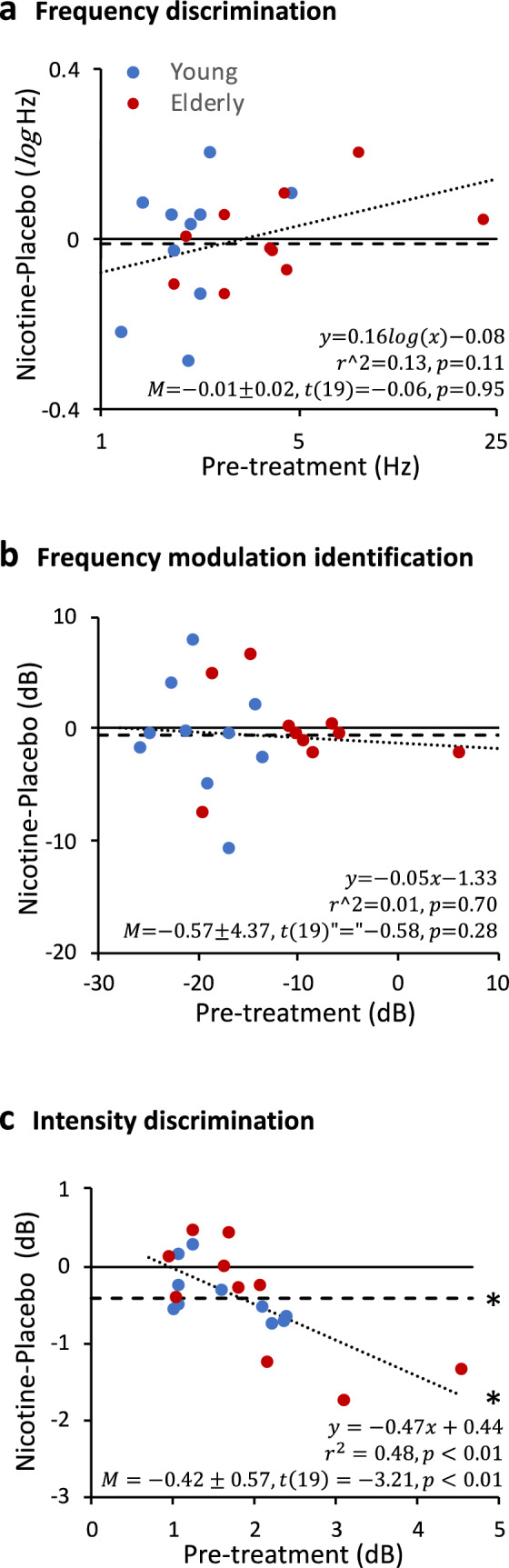
Post-treatment nicotine and placebo differences as a function of pre-treatment baseline performance in three experiments. **(a)** Frequency discrimination for young (blue circles) and elderly (red circles) adults. The baseline-dependent regression line is represented by the dotted line, with the linear regression equation being shown by text in the bottom (top line), *r*^2^ and *p*-value (second from top). The nicotine effect in terms of the mean difference between post-nicotine and post-placebo performance is represented by the dashed horizontal line, with the mean difference, standard deviation and the two-sample *t*-test result being shown by text in the bottom. The solid horizontal line crossing zero on the y-axis indicates no difference between post-nicotine and post-placebo performance. **(b)** Frequency modulation identification results are displayed following the same convention as (**a). (c)** Intensity discrimination results are displayed following the same convention as (**a).** The asterisk next to the dashed line indicates a significant difference from the baseline (solid line) and that next to the dotted indicates significant regression.

Figure 2 shows the individual differences between nicotine and placebo post-treatment performance as a function of pre-treatment baseline performance (blue circles for young individuals; red circles for elderly individuals). For frequency discrimination (Fig. 2a), there was no significant difference between nicotine and placebo post-treatment performance (the horizontal dashed line represents the mean difference: *M* = − 0.01 ± 0.02 *log*Hz, *t*(19) = 0.06, *p* = 0.95). There was no correlation between post-treatment difference and baseline performance (*r*^2^ = 0.13, *p* = 0.11).

Figure 2b shows the frequency modulation identification result. Similar to frequency discrimination, there was no significant difference between nicotine and placebo post-treatment performance [*M* = − 0.57 ± 4.37 dB, *t*(19) = − 0.58, *p* 0.05], nor any significant correlation between the post-treatment difference and the pre-treatment baseline (*r*^2^ = 0.01, *p* = 0.70).

In contrast to the results from the two frequency tasks, Fig. 2c shows that intensity discrimination produced a significant nicotine effect. First, nicotine improved intensity discrimination performance over placebo (the horizontal dashed line: *M* = − 0.42 ± 0.57 dB, *t*(19) =  − 3.21, *p* < 0.01). Second, greater improvement with nicotine treatment was correlated with worse baseline performance (the downward dotted line represents the linear regression: *r*^2^ = 0.48, *p* < 0.01). Note that the three subjects whose performance was improved the most were all from the elderly group (the three red bottom circles).

## Discussion

The present study tested two hypotheses in three auditory tasks: (1) nicotine improves auditory processing, and (2) the amount of improvement is greater in those with poorer baseline performance. The results were task-dependent. First, frequency and intensity discrimination results were consistent with the first hypothesis, but for different reasons. The nicotine effect in frequency discrimination came from improved post-nicotine performance over the pre-nicotine performance (Fig. 1a, right panel), whereas that in intensity discrimination came from improved post-nicotine performance over the post-placebo performance (Fig. 1c, right panel and Fig. 2c). Second, only the intensity discrimination result supported the second hypothesis, with the greatest improvement from those with poorer baseline performance, especially in some elderly individuals (Fig. 2c). Third, the frequency modulation identification result did not support either of these two hypotheses. We note, however, the data were trending towards the right direction, with placebo producing worse post-treatment performance and nicotine producing better post-treatment performance (Figs. 1b, 2b). The present study was underpowered to reach the level of statistical significance.

### Nicotine effects on human auditory performance

Different from relatively large and consistent nicotine effects in animal studies^7,34^, human studies, due to inherent limitations in sample selection and experimental control, often produce small and inconsistent results. For example, Harkrider and Hedrick^10^ found a significant nicotine effect on speech perception, whereas Knott et al.^11^ did not. Another example is the inconsistent effect of baseline performance on nicotine-induced improvement. Knott et al.^24,25^ found that nicotine’s effects were baseline-dependent, but Pham et al.^12^ did not observe such a dependence. Moreover, the nicotine effect may depend on the task. Pham et al.^12^ found that nicotine improved performance for auditory selective attention and tone-in-noise detection but not for easier temporal or spectral resolution tasks. Similar to the previous studies, the present study found a significant nicotine effect on intensity and frequency discrimination but not on frequency modulation identification (see “Limitations and Future Directions” below).

### Physiological mechanisms

While age-related impairments have been observed in nicotinic acetylcholine receptor signaling along the auditory pathway^19–21^, it is not clear how these impairments are related to the observed nicotine effects on auditory processing^35–38^. A particularly difficult issue to untangle is the interaction between aging and hearing loss, which may differentially influence the present auditory tasks. Frequency discrimination, even at 500 or 1000 Hz as in the present study, still relies on information from high frequencies^22^. The presence of high-frequency hearing loss (30–45 dB HL, see the Subjects section) in the elderly participants may not only produce poorer performance compared to younger participants in the two frequency tasks, but also potentially confound any age-related nicotine effect. On the other hand, intensity discrimination relies on changes in neural activities at the local frequency channel^23^. The presence of high-frequency hearing loss in the elderly participants had no effect on intensity discrimination at 500 Hz, thereby revealing a significant nicotine effect. Interestingly, similar results for frequency and intensity discrimination were also observed in individuals with tinnitus^39^, further suggesting the importance of considering both subject and task variables in investigating the effects of nicotine on auditory processing.

## Limitations and future directions

One limitation of the present study involves testing only a small number of participants, limiting its statistical power for uncovering potential effects of age and sex^11,40^. For example, the small sample size was likely responsible for a lack of a significant difference in intensity discrimination between the young and elderly adults in the present study. A second limitation is that a single dose of orally-administered nicotine may not be sufficient to effectively change perception, reflecting variability of absorption rate across participants and their different pharmacodynamic thresholds^33^. A third limitation is the choice of tasks, which were all relatively simple auditory processing tasks. Future work could improve upon these present limitations by increasing sample size, by using alternative administration routes (e.g., transdermal patch), multiple dosing levels and monitoring individual plasma level data^41^, and by targeting central auditory processing involving both temporal and spectral variance cues^42^.

## Conclusions

The present study evaluated acute effects of oral nicotine treatment on three auditory tasks in young adult and elderly, healthy, non-smoking individuals. All had normal hearing within the frequency range of the stimuli presented for the three tasks. Compared to pre-treatment performance, nicotine improved frequency discrimination. Compared to placebo, nicotine produced no overall effects on the two frequency related tasks, but significantly improved intensity discrimination, with more improvement obtained for those who had lower baseline performance. The present results support the hypothesis that nicotine enhances auditory processing, but this enhancement is task-dependent.
